# LAMP-Coupled CRISPR–Cas12a Module for Rapid and Sensitive Detection of Plant DNA Viruses

**DOI:** 10.3390/v13030466

**Published:** 2021-03-12

**Authors:** Ahmed Mahas, Norhan Hassan, Rashid Aman, Tin Marsic, Qiaochu Wang, Zahir Ali, Magdy M. Mahfouz

**Affiliations:** Laboratory for Genome Engineering and Synthetic Biology, King Abdullah University of Science and Technology (KAUST), Thuwal 23955, Saudi Arabia; ahmed.mahas@kaust.edu.sa (A.M.); norhan.hassan@kaust.edu.sa (N.H.); rashid.aman@kaust.edu.sa (R.A.); tin.marsic@kaust.edu.sa (T.M.); qiaochu.wang@kaust.edu.sa (Q.W.); zahir.ali@kaust.edu.sa (Z.A.)

**Keywords:** molecular diagnostics, plant viruses, CRISPR–Cas12, biosensing, genome engineering

## Abstract

One important factor for successful disease management is the ability to rapidly and accurately identify the causal agent. Plant viruses cause severe economic losses and pose a serious threat to sustainable agriculture. Therefore, optimization of the speed, sensitivity, feasibility, portability, and accuracy of virus detection is urgently needed. Here, we developed a clustered regularly interspaced short palindromic repeats (CRISPR)-based nucleic acid diagnostic method utilizing the CRISPR–Cas12a system for detecting two geminiviruses, tomato yellow leaf curl virus (TYLCV) and tomato leaf curl New Delhi virus (ToLCNDV), which have single-stranded DNA genomes. Our assay detected TYLCV and ToLCNDV in infected plants with high sensitivity and specificity. Our newly developed assay can be performed in ~1 h and provides easy-to-interpret visual readouts using a simple, low-cost fluorescence visualizer, making it suitable for point-of-use applications.

## 1. Introduction

Plant viruses are responsible for many commercially important plant diseases, infecting a wide range of plant species and resulting in severe quality and yield losses in diverse crops. Plant diseases are estimated to cause 10 to 15% reductions in global crop yields annually, with 47% of these losses caused by viruses [1], including geminiviruses. Geminiviruses have circular single-stranded DNA (ssDNA) genomes, replicate in the nuclei of plant cells, and are destructive plant pathogens that reduce yields in vegetables, grains, and fruit crops worldwide. For example, geminiviruses infect and seriously damage many dicotyledonous crop plants, including tomato (*Solanum lycopersicum*), cotton (*Gossypium* sp.), cassava (*Manihot esculenta*), sugar beet (*Beta vulgaris*), and pepper (*Capsicum annuum*) [2,3].

Methods for the timely and accurate identification of the causal agents of viral diseases are urgently needed for effective disease management and eradication. Several diagnostic methods for plant viruses have been developed [4]. However, these methods suffer from various limitations, including the required time, equipment, and expertise, as well as the high rate of inaccurate results, which complicate their use for the rapid, simple identification of plant viruses [5]. 

Clustered regularly interspaced short palindromic repeats (CRISPR)-based platforms are excellent for developing sensing tools to detect viruses and other pathogens [6,7]. Since the introduction of CRISPR-based gene editing, an array of innovative diagnostics relying on CRISPR–Cas systems have been used to detect various pathogens with unprecedented speed and accuracy, as represented by the DNA endonuclease-targeted CRISPR trans reporter (DETECTR) [8] and specific high-sensitivity enzymatic reporter unlocking (SHERLOCK) systems [9]. These CRISPR-based diagnostics involve the isothermal amplification of a target sequence, followed by target recognition via CRISPR–Cas proteins (such as Cas12 in DETECTR or Cas13 in SHERLOCK) and the collateral cleavage of a DNA or RNA reporter to indicate the presence of the target [10]. Despite its widespread use for the detection of various human and animal pathogens [7,11], the application of CRISPR for identifying plant DNA viruses has been limited. 

**Figure 1:**
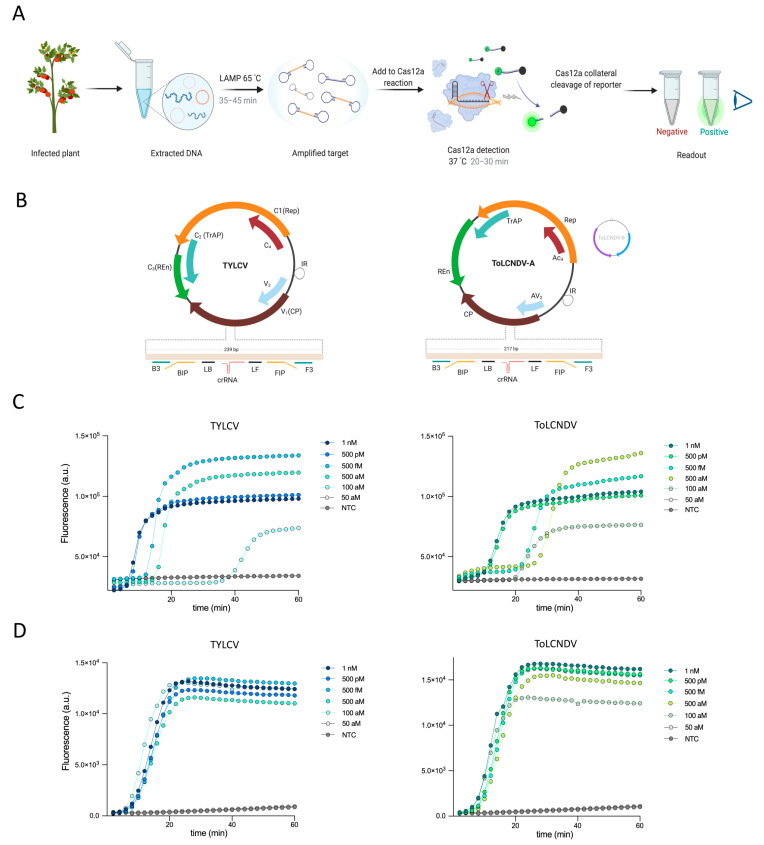
LAMP-coupled Cas12-based assay for the detection of TYLCV and ToLCNDV. (**A**) The assay workflow. Viral DNA (orange circles) extracted from an infected tomato plant is amplified by loop-mediated isothermal amplification (LAMP), followed by clustered regularly interspaced short palindromic repeats (CRISPR)-mediated detection. Cas12a-based detection of the LAMP product triggers collateral cleavage of the reporter, thus producing a signal for visual detection. (**B**) Organization of the single-component TYLCV genome (left) and the two-component (bipartite) ToLCNDV-A and B genomes (right). The targeted areas in the coat protein gene (*CP*) are highlighted, with the designed LAMP primers and crRNAs shown. Rep: replication-associated protein; IR: intergenic region; CP: coat protein; REn: replication enhancer protein; TrAP: transcriptional activator protein; AC4 or C4: RNA suppressor protein, present on the antisense (complementary) strand; AV2 or V2: precoat proteins, present on the virion-sense strand as plant RNA silencing suppressors. (**C**) Monitoring the performance of LAMP of synthetic DNA generated by PCR of TYLCV dsDNA (left) and ToLCNDV dsDNA (right) by real-time fluorescence across a range of dsDNA concentrations at 65 °C for 60 min. The LAMP signal was measured using the nucleic acid stain SYTO 9. Data generated using 50 aM of sample showed fluorescence signals similar to those of the no-template control (NTC), and these lines are therefore overlapping. Data are shown as the mean (*n* = 3). (**D**) Real-time measurements of Cas12 collateral activity on HEX reporter with LAMP-amplified DNA from a synthetic dsDNA template. Cas12a with crRNA targeting TYLCV (left) or ToLCNDV (right) was incubated with HEX reporter and LAMP product at 37 °C for 60 min. Data generated using 50 aM of sample showed fluorescence signals similar to those of the NTC, and these lines are therefore overlapping. Data are shown as the mean (*n* = 3).

Here, we report the development and validation of a specific, sensitive CRISPR–Cas12-based assay for detecting plant geminiviruses. This assay involves the sensitive, specific amplification of viral DNA sequences isolated from infected plants using loop-mediated isothermal amplification (LAMP), followed by the detection of the target sequence and cleavage of the fluorescence reporter by Cas12, which indicates that the viral sequence has been detected. Our assay generates easy-to-interpret visual readouts using a simple, low-cost handheld visualizer, suggesting that it could be performed in the field (Figure 1A).

## 2. Results and Discussion

We developed our assay to detect two geminiviruses: tomato yellow leaf curl virus (TYLCV) (monopartite begomovirus) and tomato leaf curl New Delhi virus (ToLCNDV) (bipartite begomovirus). Both viruses belong to the family Geminiviridae, genus *Begomovirus*; these are two of the most destructive viruses in various crops, especially tomato [12]. To design LAMP primers, we identified conserved regions of the TYLCV and ToLCNDV genomes, respectively, via sequence alignment of various strains from the National Center for Biotechnology Information (NCBI). Our analysis revealed highly conserved regions within the coat protein (*CP*) gene sequences of both viruses. This feature allowed us to design LAMP primers to amplify these conserved regions and efficient Cas12a crRNAs targeting the resulting LAMP amplicon with an appropriate Cas12a protospacer adjacent motif (PAM) sequence (5′-TTTN-3′) for Cas12-mediated detection within the ToLCNDV-A genomic component, as well as the single genomic component of TYLCV (Figure 1B) (Supplementary Table S1). 

LAMP is the first step in our assay for viral sequence detection. We first determined the sensitivity of our assay by evaluating the performance of LAMP using synthetic TYLCV and ToLCNDV double-stranded DNAs (dsDNAs) as input for real-time LAMP. Synthetic DNA at concentrations as low as 100 aM was detected after ~45 min of LAMP with dsDNA from TYLCV and after ~35–40 min with dsDNA from ToLCNDV, whereas no amplification product was detected in reactions with no template (no-template control, NTC) (Figure 1C).

Although various isothermal amplification-based approaches have been developed for plant virus detection, the high rate of nonspecific amplification and high vulnerability to contamination associated with these isothermal amplification methods limit their use for the detection of specific viruses [13]. Therefore, due to the high specificity of CRISPR–Cas systems, we sought to couple LAMP with Cas12a-based detection for the sensitive, highly specific detection of amplified viral sequences. Since Cas12a performs optimally at 37 °C, while LAMP is performed at 65 °C, our assay involves two steps: LAMP at 65 °C, followed by Cas12a-based detection of the viral sequence at 37 °C.

Using real-time fluorescent readouts, we evaluated the sensitivity of Cas12a-based detection of LAMP-amplified virus sequences. LAMP-coupled Cas12 was able to detect as little as 100 aM of synthetic dsDNA, whereas no significant signal was observed in the NTC (Figure 1D). Next, since host plants are commonly infected with multiple viruses, we tested for cross-reactivity of our TYLCV or ToLCNDV detection assay with other common geminiviruses using plasmid DNA as an input, including merremia mosaic virus (MeMV), tobacco leaf curl virus (TLCV), pedilanthus leaf curl virus (PeLCV), and cotton leaf curl Kokhran virus (CLCuKV). Our assay revealed no cross-reactivity with any of these viruses, pointing to the high specificity of our assays (Supplementary Figure S1). Together, our observations demonstrated that our LAMP primers and Cas12a crRNAs efficiently, sensitively, and specifically amplify and detect low concentrations of viral sequences; 40–45 min of LAMP followed by 25–30 min of Cas12a-based detection was sufficient for the sensitive detection of low concentrations of viral sequences, allowing our assay to be completed within ~1 h. 

**Figure 2:**
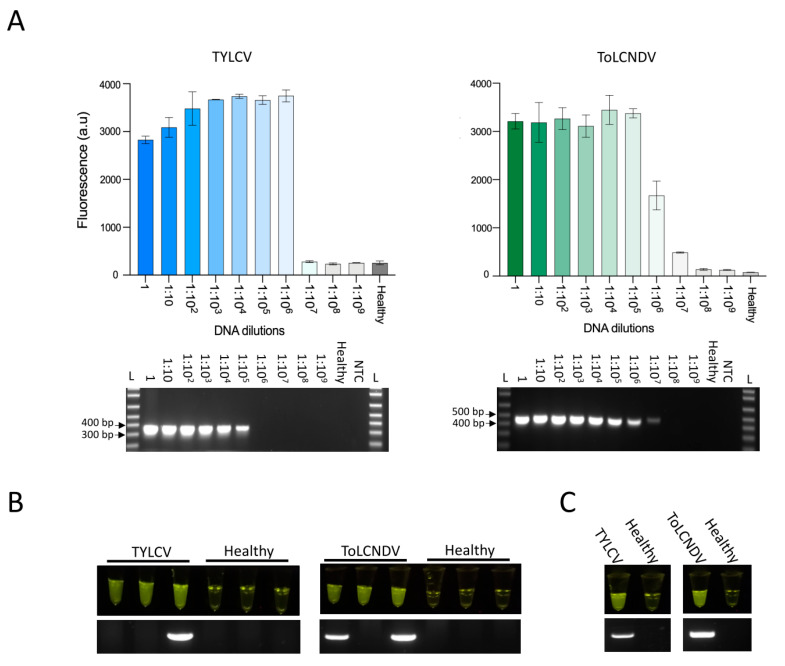
Detection of TYLCV and ToLCNDV in infected plants. (**A**) Comparison of Cas12-based detection (top) and conventional PCR detection (bottom) of 10 serial dilutions of TYLCV and ToLCNDV DNA from infected *Nicotiana benthamiana* plants. Different dilutions of DNA extracted from plants infected with TYLCV (left) or ToLCNDV (right) or noninfected plants (healthy) were used as input for the LAMP reactions at 65 °C for 40 min as well as PCR. LAMP products were subsequently added to the Cas12a detection reactions. The Cas12a detection assay was performed at 37 °C, and collateral activity was measured by HEX reporter fluorescence after 30 min. Data are shown as mean ± SD (*n* = 3). Conventional PCR products were resolved on a 1% agarose gel. L: 1 kb plus ladder (Invitrogen). (**B**) Comparison of Cas12a-based virus detection with visual in-tube fluorescence readouts (top) and conventional PCR (bottom) of three independent *N. benthamiana* plants infected with TYLCV (left panel) or ToLCNDV (right panel) or noninfected plants (healthy). (**C**) Comparison of Cas12a-based virus detection with visual in-tube fluorescence readouts (top) and conventional PCR (bottom) of tomato plants infected with TYLCV (left panel) or ToLCNDV (right panel) or noninfected plants (healthy).

Next, we employed our assay to detect viral sequences in infected plants. First, we tried to detect viral sequences from DNA extracted from the upper leaves of *Nicotiana benthamiana* plants subjected to *Agrobacterium*-mediated infection with TYLCV or ToLCNDV. We confirmed that the *N. benthamiana* plants were infected with these viruses by conventional PCR using primers specific to TYLCV and ToLCNDV (Supplementary Figure S2). We then compared the performance of our LAMP–Cas12a-based assay with that of conventional PCR to detect viral sequences from DNA extracted from infected *N. benthamiana* plants using 10-fold serial dilutions of the extracted DNA to mimic the different viral loads that could be found in different infected plants. The sensitivity and specificity of our assay were comparable to those of conventional PCR for detecting viruses at low dilution levels, with better performance obtained for the detection of TYLCV (Figure 2A). 

To generate a simple readout and facilitate the interpretation of the results, we developed a field-deployable rapid system that does not require complex equipment by generating an in-tube fluorescent readout using the same HEX fluorescence reporter used in the above experiments. In this system, the release of the HEX fluorophore in the reporter molecule via collateral Cas12a activity generates a bright fluorescent signal when excited by light-emitted diode (LED); this fluorescence can easily be visualized using a low-cost, simple handheld P51 Molecular Fluorescence Viewer. We tested our detection method using *N. benthamiana* plants infected with TYLCV or ToLCNDV or healthy (noninfected) plants. We performed a head-to-head comparison of our assay with conventional PCR analysis for the detection of both viruses. Our in-tube fluorescent readout-based assay produced positive results for all TYLCV- and ToLCNDV-infected *N. benthamiana* plants and negative results for all healthy noninfected plants (Figure 2B). By contrast, although the results of PCR analysis were negative for all healthy plants, this technique consistently failed to detect some of the TYLCV- or ToLCNDV-infected plants, highlighting the high sensitivity of our assay (Figure 2B). The enhanced sensitivity of our LAMP-coupled Cas12a detection assay over PCR analysis is likely due to the high sensitivity of LAMP, which has been shown to have superior sensitivity compared with other PCR-based amplification techniques for the diagnosis of different pathogens [14].

Finally, we assessed whether we could detect TYLCV and ToLCNDV in tomato plants, as tomato is a major host plant of these viruses. DNAs extracted from tomato plants infected with TYLCV, ToLCNDV, or healthy plants were used for our LAMP-coupled Cas12a detection assay. Our assay unambiguously and accurately detected both viruses using LAMP primers and Cas12a targeted to its cognate viral sequence, whereas no positive readouts were obtained using noninfected plants. These results are in total agreement with the results of conventional PCR analysis (Figure 2C). These results help confirm our findings for *N. benthamiana* plants and demonstrate the utility of our assay for detecting DNA viruses in crop plants. 

In summary, we developed a new platform for the rapid, sensitive, and specific detection of plant DNA viruses. Our newly developed detection method has significant advantages over the currently available tools for plant DNA virus diagnosis, including its ease of use, speed, and low cost. Although there have been great advances in the development of platforms for plant virus diagnosis, including serological and molecular techniques (especially isothermal amplification methods), several drawbacks still limit their use, including their low sensitivity and specificity, the complexity of the reactions, and the time required [5]. There have been impressive developments in CRISPR-based diagnostics for detecting various pathogens, including the novel SARS-CoV-2 virus [15,16]. However, despite the various applications of CRISPR technology in plant virology [17,18,19,20,21,22], CRISPR-based diagnostic techniques are rarely used for plant pathogens. A recent study demonstrated the successful use of CRISPR-based diagnostics for the sensitive, specific detection of plant RNA viruses and viroids in apple (*Malus domestica*) [23]. This technique and the current platform lay the foundation for further development of CRISPR-based diagnostics for the rapid, sensitive, specific detection of (in principle) all plant pathogens. Our platform generates simple, easy-to-interpret visual readouts within 1 h, suggesting that it could be suitable for point-of-use diagnostic applications. Several studies have demonstrated the direct LAMP-based amplification of viral sequences from crude extracts [13]. Therefore, further improvement of our current LAMP-coupled Cas12a method could facilitate the development of our assay as an in-field diagnostic test. In conclusion, our findings demonstrate that our LAMP-coupled Cas12a method is reliable for the rapid diagnosis of plant DNA viruses and could be useful for in-field diagnostics.

## Data Availability

The data presented in this study are available in supplementary material file.

## Supplementary Materials

The following are available online at https://www.mdpi.com/1999-4915/13/3/466/s1: Figure S1: Test of cross-reactivity with other DNA viruses, Figure S2: Examining virus infectivity in N. benthamiana plants; Table S1: TYLCV and ToLCNDV LAMP primers and Cas12a crRNAs.
