# Supplementary material for: LAMP-Coupled CRISPR–Cas12a Module for Rapid and Sensitive Detection of Plant DNA Viruses
Source: Viruses. 2021 Mar 12;13(3):466. doi: 10.3390/v13030466 (PMC8001329; doi:10.3390/v13030466)
Supplement: Supplementary file 1 [file viruses-13-00466-s001.pdf]

SUPPLEMENTARY FILE

LAMP-coupled CRISPR-Cas12a module for rapid and sensitive detection of plant DNA viruses

Ahmed Mahas[†], Norhan Hassan[†], Rashid Aman, Tin Marsic, Qiaochu Wang, Zahir Ali and [*]Magdy M. Mahfouz

[1]Laboratory for Genome Engineering and Synthetic Biology, King Abdullah University of Science and Technology (KAUST), Thuwal, Saudi Arabia.

†Joint first authors

[*]Correspondence to Magdy Mahfouz (Magdy.mahfouz@kaust.edu.sa)

Supplementary Figures:

Supplementary Figure S1: Test of cross-reactivity with other DNA viruses

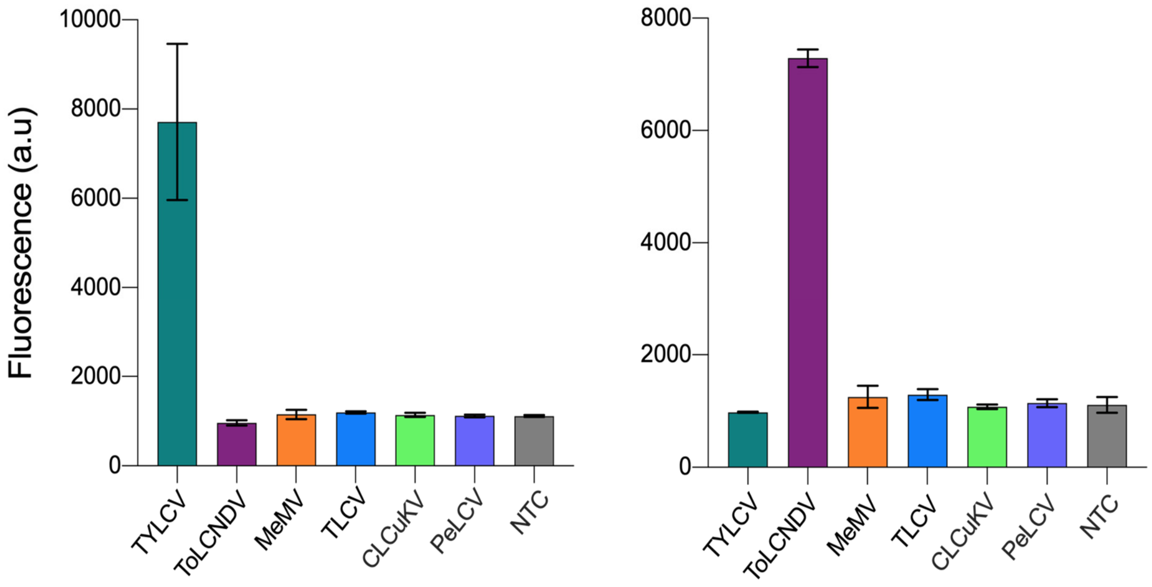

Supplementary Figure S1: Test of cross-reactivity with other DNA viruses. To test for cross-reactivity, TYLCV, ToLCNDV-A, MeMV, TLCV, CLCuKV, or PeLCV DNA plasmids were used as input for a LAMP-coupled Cas12a assay for detection of TYLCV (left) or ToLCNDV (right). Cas12a collateral activity was measured as end-point detection of HEX reporter fluorescence after 30 min. Data are shown as mean ± SD (N=2). NTC: no template control.

Supplementary Figure S2: Examining virus infectivity in *N. benthamiana* plants.

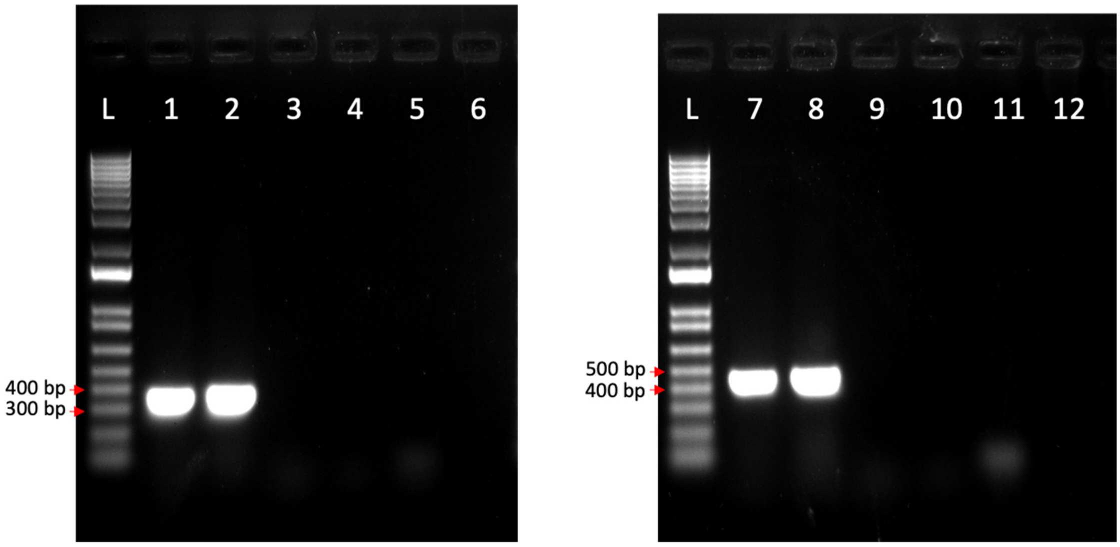

Supplementary Figure S2: Examining virus infectivity in *N. benthamiana* plants. PCR amplicons resolved on 1% agarose gels. Left panel, PCR results with primers specific to the TYLCV genome. Lane 1 and 2 are two replicates of PCR with DNA extracted from *N. benthamiana* plants infected with TYLCV, lane 3 and 4 with DNA extracted from *N. benthamiana* plants infected with ToLCNDV, and lane 5 and 6 with DNA extracted from healthy (non-infected) *N. benthamiana* plants. Right panel, PCR results with primers specific to the ToLCNDV genome. Lane 7 and 8 are two replicates with DNA extracted from *N. benthamiana* plants infected with ToLCNDV, lane 9 and 10 with DNA extracted from *N. benthamiana* plants infected with TYLCV, and lane 11 and 12 with DNA extracted from healthy (non-infected) *N. benthamiana* plants. L: 1 kb plus ladder (Invitrogen).

Supplementary Table S1: TYLCV and ToLCNDV LAMP primers and Cas12a crRNAs.

Oligo name	Sequence (5`->3`)
TYLCV-F3	GGTAAAGTCTGGATGGATGA
TYLCV-FIP	ACCTGTCCAAAATCCATTGGCAGAATCACACTAATCAGGTC
TYLCV-LF	CTATCACGGACCAAGAAGAAC
TYLCV-B3	TGTTCCTTCATTCCAGAGG
TYLCV-BIP	CAGTACCGCAACCGTGAAGACAATAACTGTAGCATGAAATTTCCT
TYLCV-LB	GATTTGCGGGATAGGTTTCAAG
ToLCNDv-F3	GCCCATGAACAGAAAACC
ToLCNDv-B3	TTTTCCCAGTACATAGACAGA
ToLCNDv-FIP	CGATTGCACCTTACAAGGGCTGTACAGAATGTATAGAAGTCCT
ToLCNDv-BIP	CTCGCATATTGGCAAAGTCATGTCACAAAATCGCTTGCCTAC
ToLCNDv-LF	TCACAACCCCTTGGCACG
ToLCNDv-LB	GTGATGTTACTCGAGGCAC
Synthetic PCR-TYLCV-F	GATGTCGAAGCGACCAGGCGATATAATC
Synthetic PCR-TYLCV-R	GCGTATTTTCATAGTTGCATACACTG
Synthetic PCR-ToLCNDV-F	ATTTCAACTCCCGCATCG
Synthetic PCR-ToLCNDV-R	AATAGATCCGGATTTTCAAAGT
Convectional PCR-TYLCV-F	AGGTCAGCACATTTCCATCC
Convectional PCR-TYLCV-R	GTTCTCGGACACCCATACTTC
Convectional PCR-ToLCNDV-F	GTCTCCGCATATCCATGTTCTC
Convectional PCR-ToLCNDV-R	AGCGGAACACACCACATTAC
T7- Forward primer	GAAATTAATACGACTCACTATAGGG
TYLCV-Cas12a crRNA	GGGCTCATTATCGAACATATATCTACACTTAGTAGAAATTACCCTA TAGTGAGTCGTATTAATTTC
ToLCNDv-Cas12a crRNA	CGAGACATCGTGCCGGGATTATCTACACTTAGTAGAAATTACCCT ATAGTGAGTCGTATTAATTTC
HEX reporter	HEX-TTATTATT-3IABkFQ

Materials and Methods

Design and screening of primers

Multiple virus genome sequences of different strains and isolates obtained from the NCBI database were aligned separately for both TYLCV and ToLCNDV with ClustalW using Unipro UGENE software, which showed high conservation of different genomic regions. Full length of genomic sequences from different isolates of the common TYLCV strains, including TYLCV-Mid and TYLCV-IL (GenBank Accession Numbers: JX128100.1, LC202091.1, KC106651.1, AY044138.1, KX347166.1, DQ644565.1, EF539831.1, AB636264.1) and the common Spanish (ES) ToLCNDV strain (GenBank Accession Numbers: KF749224.1, KF891468.1, KM977733.1, KF749223.1, KT175406.1) were used for the alignment. Different primer sets targeting a conserved sequence in the coat protein genes in both viruses were designed using PrimerExplorer v5 (https://primerexplorer.jp/e/). Primer sets that showed the best performance were identified by conducting LAMP assays to detect specific targets as described below. LbCas12a crRNAs were designed manually to target regions amplified by LAMP and containing the required LbCas12a PAM sequences.

Protein purification

LbCas12a recombinant proteins were purified as described by Chen *et. al* [1].

Nucleic acid preparation

Synthetic virus-containing dsDNA was obtained by PCR amplification of the specific target using PCR primers targeting the TYLCV plasmid that was available in the laboratory [2] and the

ToLCNDV plasmid kindly provided by Dr. José-Antonio Daròs. The primer sequences used for TYLCV and ToLCNDV are shown in (Supplementary Table 1).

The PCR products were gel purified using a QIAquick Gel Extraction kit following the manufacturer's instructions. This PCR product was used to screen the different sets of LAMP primers and to assay for LAMP sensitivity.

For LbCas12a crRNAs, templates for *in vitro* transcription were generated using single-stranded DNA oligos containing a T7 promoter, scaffold and spacer in reverse complementary orientation (IDT), which were then annealed to the T7 forward primer (Supplementary Table 1) in Taq DNA polymerase buffer (Invitrogen). The *in vitro* transcribed sgRNAs were purified using a MEGAclear Transcription Clean-Up Kit (Thermo Scientific AM1908) following the manufacturer's instructions.

Loop-mediated isothermal amplification (LAMP)

The detection assays were performed using two steps. In the first step, LAMP was performed to amplify the viral DNA to generate dsDNA substrates for Cas12 enzymes. All of the reagents for the LAMP reactions were assembled on ice and combined in a single reaction mixture using a commercial NEB WarmStart LAMP Kit (M1700S) as per the manufacturer's protocol, where we added 1 µL 10X Primer Mix (2 µM F3 2 µM B3 16 µM FIP 16 µM BIP 8 µM LF 8 µM LB) (Supplementary Table 1), 2 µL of viral (control or extracted DNA), 5 µL WarmStart LAMP 2X master mix and nuclease-free water up to 10 µL. The mixtures were incubated at 65°C for 30, 45, and 60 minutes, or as indicated.

Real-time LAMP reactions were performed as described above with the addition of 250 nM SYTO-9 Fluorescent Nucleic Acid Stain (ThermoFisher S34854) with different

concentrations of synthetic viral DNA in 10-µL reactions with three technical replicates in 96-well format and read out using a StepOnePlus Real-Time PCR System (Applied Biosystems) with fluorescent measurements performed every 2 minutes.

Cas12a-based detection

For the Cas12-based detection assays, 250 nM LbCas12a was pre-incubated with 250 nM of specific LbCas12a crRNAs in 1x Cas12 reaction buffer (20 mM Tris-HCl, pH 7.5, 100 mM KCl, 5 mM $MgCl_2$, 1 mM DTT, 5% glycerol, 50 µg ml^{-1} heparin) for 30 min at 37°C to assemble Cas12-crRNA ribonucleoprotein (RNP) complexes. The RNP mixture was diluted 4 times with 1x cleavage buffer (20 mM HEPES [pH 7.5], 150 mM KCl, 10 mM $MgCl_2$, 1% glycerol, and 0.5 mM DTT). For fluorescence-based detection, 2 µL of LAMP product was added to 750 nM of HEX reporter (IDT), mixed with 50 nM of pre-assembled Cas12-sgRNA RNP complexes in a 23-µL reaction and incubated at 37°C for 30 min. End-point visual detection was monitored using a P51 Molecular Fluorescence Viewer (https://www.minipcr.com/product/p51-molecular-glow-lab/) in a dark room, and photographs were taken using a smart phone with normal settings. For real-time detection of Cas12a-induced collateral cleavage of the HEX reporter, the Cas12 detection reaction was loaded into a 384 Nunc white plate and loaded into a Tecan plate reader (Tecan M 1000 Pro) pre-heated to 37°C, and fluorescence measurements were taken every 2 minutes for 60 min.

Agroinfiltration and sap inoculation of plants

Infectious clones of the DNA viruses, including TYLCV2.3 [2], ToLCNDV-A, and ToLCNDV-B (kindly provided by Dr. José-Antonio Daròs) were individually electroporated into *A.*

tumefaciens strain GV3101. Single colonies were grown overnight in selective medium, centrifuged, suspended in infiltration medium (10 mM MES [pH 5.7], 10 mM $MgCl_2$, and 200 μM acetosyringone) and incubated at ambient temperature for 2 h. To infiltrate the leaves of *N. benthamiana* plants, cultures of TYLCV or ToLCNDV-A and -B were mixed at a final OD_{600} of 0.5. In case of ToLCNDV, the two bacterial cultures (ToLCNDV-A and ToLCNDV-B) were mixed at a ratio of 1:1. Healthy, fully developed leaves of ~ 3-week-old *N. benthamiana* plants were selected for the experiments, and agroinfiltration was performed with a 1-mL needleless syringe into the lower side of the selected leaves. Samples (systemic leaves) were collected ~ 10 days after infiltration (dai).

To infect tomato (*Solanum lycopersicum*) plants, systemic young leaves of previously infected *N. benthamiana* plants (10 dai) were ground to a fine powder in liquid nitrogen and re-suspended in 1: 4 w/v potassium phosphate buffer (0.1 M, pH 8.0). The surfaces of three leaves of a tomato plant were dusted with carborundum (200–450 mesh). Sap was applied to the entire adaxial surface by thoroughly rubbing with a sap-dipped pestle in a circular motion. Systemic leaves were collected at 21 days after sap application and subjected to DNA extraction as described below.

Sample collection and DNA extraction

Leaves of infected plants were collected, ground to a fine powder in liquid nitrogen with a mortar and pestle, and total DNA extracted from ~100 mg of sample using the Tiangen DNAquick Plant System following the manufacturer's protocol.

Virus infection confirmation using conventional PCR

To confirm the presence of TYLCV and ToLCNDV in infected plants, PCR was performed with the virus-specific primers (Supplementary Table 1) using Phusion polymerase (NEB M0530L) following the manufacturer's protocol.

References

1. Chen, J.S.; Ma, E.; Harrington, L.B.; Da Costa, M.; Tian, X.; Palefsky, J.M.; Doudna, J.A. CRISPR-Cas12a target binding unleashes indiscriminate single-stranded DNase activity. *Science* **2018**, *360*, 436-439, doi:10.1126/science.aar6245.
2. Ali, Z.; Abulfaraj, A.; Idris, A.; Ali, S.; Tashkandi, M.; Mahfouz, M.M. CRISPR/Cas9-mediated viral interference in plants. *Genome Biology* **2015**, *16*, 238, doi:10.1186/s13059-015-0799-6.
